# Case Report: Pathologic complete response in triple negative breast cancer treated with anthracycline free regimen

**DOI:** 10.3389/fonc.2026.1733621

**Published:** 2026-01-30

**Authors:** Francesca Piazza, Giulia Scartabellati, Laura Moretti, Marta Laganà, Lucia Vassalli, Benedetta Trevisan, Michela Bazzoli, Greta Schivardi, Federico Canzi, Roberto Baraziol, Giuseppe Ippolito, Salvatore Grisanti, Alfredo Berruti, Deborah Cosentini, Rebecca Pedersini

**Affiliations:** 1 Department of Medical and Surgical Specialties, Radiological Sciences and Public Health, Medical Oncology, University of Brescia, Azienda socio-sanitaria territoriale (ASST) Spedali Civili, Brescia, Italy; 2 Division of Surgery, Azienda socio-sanitaria territoriale (ASST) Spedali Civili, Brescia, Italy; 3 Division of Plastic and Reconstructive Surgery, Azienda socio-sanitaria territoriale (ASST) Spedali Civili, Brescia, Italy; 4 Struttura Semplice Dipartimentale (SSD) Breast Unit, Azienda socio-sanitaria territoriale (ASST) Spedali Civili of Brescia, Brescia, Italy

**Keywords:** neoadjuvant treatment, TNBC, keynote 522, pathologic complete response, side effects, de-escalation, pCR

## Abstract

Triple-negative breast cancer (TNBC) is a highly aggressive malignancy with limited therapeutic options and elevated mortality rates. Chemo-immunotherapy according to the KEYNOTE-522 has established a new standard of care in the neoadjuvant setting, due to high rates of pathological complete response (pCR) and improved survival outcomes. Recent evidence suggests the feasibility of treatment de-escalation, particularly the omission of anthracyclines, to mitigate treatment-related toxicity; however, this approach is yet to be established in clinical practice. We report the clinical case of a complete pathological response despite the omission of anthracyclines in a patient with high-risk TNBC. A 54-year-old Caucasian woman was diagnosed in February 2024 with cT4a cN0 TNBC and started neoadjuvant treatment according to the KEYNOTE-522 regimen. Radiologic mid-treatment evaluation showed a marked reduction in tumor size (22 mm vs 78 mm), supporting continuation to the second treatment phase. However, epirubicin extravasation required interruption of treatment, and prompt management of the risk of infection and necrosis was necessary. Once the risk of sepsis was ruled out, the patient underwent surgery, achieving pCR (ypT0, ypN0). This case supports the potential role of de-escalated regimen in selected patients and raises the hypothesis-generating question of whether the inflammatory response triggered by the extravasation of epirubicin has enhanced the immune-mediated anti-tumor effect, potentially making pembrolizumab more effective. The lack of validate predictive biomarkers for patient selection and the absence of reliable imaging techniques to predict pathologic complete response are also discussed. Tumor-infiltrating lymphocytes (TILs) are currently the only biomarkers consistently shown to predict response to neoadjuvant treatment; however, the low TIL level observed in our patient (5%) highlights that this parameter is still far from being a reliable tool to guide treatment de-escalation in clinical practice. Further investigation is warranted to identify which patients could safely benefit from reduced-intensity approaches without compromising outcomes and how clinicians can be guided in this decision-making process.

## Introduction

Triple-negative breast cancer (TNBC) is defined by the absence of expression of oestrogen (ER) and progesterone (PR) receptors, and human epidermal growth factor receptor 2 (HER2) overexpression and/or gene amplification. It accounts for up to 15% of all breast malignancies and, despite early-stage diagnosis, TNBC is often characterized by a high propensity for early recurrence and elevated mortality rates (1).

The KEYNOTE-522 study has established a new standard of care for an increasing subset of patients with stage II or III TNBC (2–4). Indeed, the addition of pembrolizumab to neoadjuvant chemotherapy with carboplatin/paclitaxel followed by anthracycline-based chemotherapy (AC/EC) resulted in a significant improvement in pathological complete response (pCR) rates, increasing from 51.2% to 64.8%, with an absolute difference of 13.6% (p<0.001). Moreover, the addition of immunotherapy conferred a clinically meaningful benefit in long-term outcomes, with a 36-month event-free survival (EFS) rate of 84% in the pembrolizumab group compared to 77% in the placebo group, corresponding to a 37% reduction in the risk of disease recurrence or progression (HR 0.63, 95% CI 0.48–0.82) (3). Additionally, five-year overall survival (OS) was significantly improved, reaching 86.6% (95% CI, 84.0–88.8) with pembrolizumab versus 81.7% (95% CI, 77.5–85.2) in the placebo arm, further reinforcing the long-term efficacy of immune checkpoint inhibition in the early-stage TNBC setting (4).

After a median follow-up of 75.1 months, the majority of treatment-related adverse events were observed during the neoadjuvant phase and were predominantly associated with chemotherapy (4). The most frequently reported toxicities included nausea, vomiting, fatigue, neutropenia, febrile neutropenia, and cardiac disorders, which were possibly attributable to the administration of anthracyclines and cyclophosphamide (4). These agents are known to be associated with severe long-term toxicities, including cardiotoxicity, secondary malignancies, and infertility (5, 6).

Considering the efficacy and safety scenario described so far, it is essential to explore whether and when treatment de-escalation may be feasible in order to mitigate toxicity. However, the KEYNOTE-522 trial was not designed to evaluate the impact of adjuvant pembrolizumab or the feasibility of chemotherapy de-escalation, particularly in patients achieving pCR, leaving these questions unresolved.

To tailor therapy to individual patient needs, it would be crucial to incorporate patient age, comorbidities, clinical characteristics, and biomarker profiles into a personalized treatment approach. The absence of reliable biomarkers to identify TNBC patients who would derive the greatest benefit from immune checkpoint inhibitors (ICIs) underscores the urgent need for predictive strategies to optimize patient selection and treatment personalization.

We report a clinical case of a patient with early-stage, high-risk TNBC who achieved both clinical and pathological complete response despite the omission of four cycles of anthracyclines, cyclophosphamide, and pembrolizumab. This case prompts a discussion on the potential role of treatment de-escalation strategies in selected patient populations.

## Case report

In February 2024, a 54-year-old Caucasian woman detected a palpable mass in the right breast during self-examination. Initial breast ultrasound (US) and mammography, followed by magnetic resonance imaging (MRI), identified a neoplastic lesion measuring 78 × 53 × 68 mm in the lower outer quadrant of the right breast, with infiltration of the pectoralis muscle and extension into the chest wall. No pathological lymphadenopathies were observed.

Core needle biopsies and histological analysis confirmed a high-grade, poorly differentiated infiltrating ductal carcinoma. Immunohistochemistry (IHC) revealed the absence of oestrogen and progesterone receptors (0% and <1%, respectively) and no HER2 overexpression (IHC score 0). The tumor displayed a high proliferative index (Ki67 = 65%) and low tumor-infiltrating lymphocytes (TILs) with a score of 5%. Further assessment with a computed tomography (CT) scan of the brain, chest, abdomen, and pelvis showed no distant metastasis.

The tumor was staged as cT4a cN0 cM0, and histologically classified as TNBC. Therefore, considering the patient’s overall health status, disease extent, and risk profile, in April 2024, the multidisciplinary team recommended neoadjuvant treatment according to the Keynote-522 regimen.

After 24 weeks, the patient had completed four cycles of weekly carboplatin, paclitaxel and pembrolizumab without any significant adverse toxicity. The interim breast US demonstrated a substantial reduction in tumor size (22 mm compared to the initial 78 mm). Given this remarkable response, in August 2024, the patient transitioned to the second phase of neoadjuvant chemotherapy.

**Figure 1:**
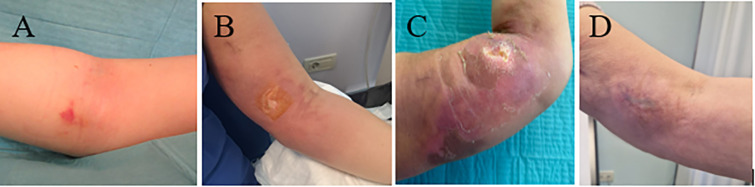
Clinical evolution of anthracycline extravasation injury of the left forearm. **(A)** Initial erythema and edema at the site of extravasation (Day 0). **(B)** Development of skin ulceration (Day 8). **(C)** Extensive tissue necrosis during the subacute phase (Week 6). **(D)** Healed lesion with residual skin changes at follow-up (Month 7).

However, shortly after the initiation of the first infusion, epirubicin extravasation occurred from the peripherally inserted central catheter-PORT (PICC-port). The extravasated drug was promptly removed mechanically, followed by topical application of dimethyl sulfoxide (DMSO). Additionally, dexrazoxane was administered over three consecutive days to mitigate tissue damage. The tissue damage progressively worsened over days to weeks, resulting in pain, thrombosis, oedema and functional impairment. (**Figures 1A–D**).

**Figure 2:**
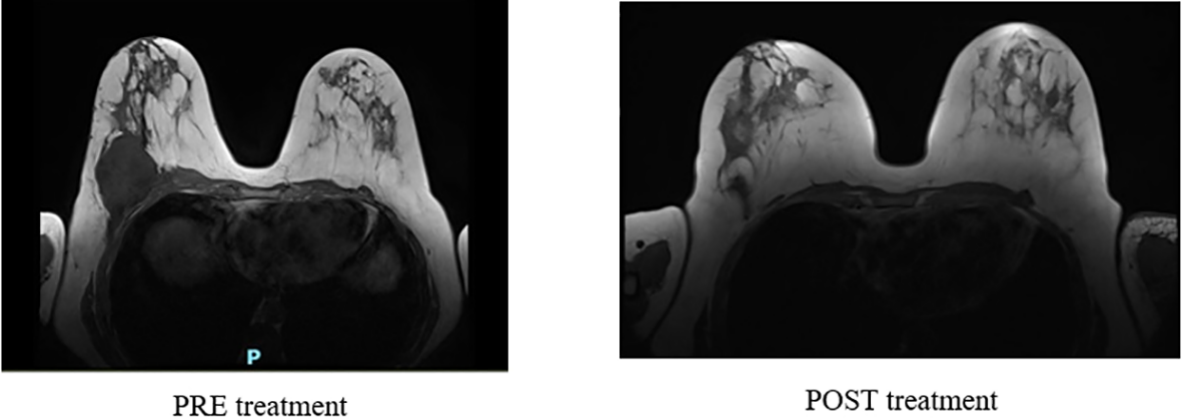
Breast MRI performed before and after neoadjuvant treatment.

The case was discussed within a multidisciplinary team, which concluded that, given the risk of infection, potentially exacerbated by chemotherapy-induced neutropenia, and the favourable objective response, neoadjuvant treatment should be discontinued, and the patient should be addressed to surgery. A breast MRI, performed before surgery, showed a radiological complete response (**Figure 2**).

**Figure 3:**
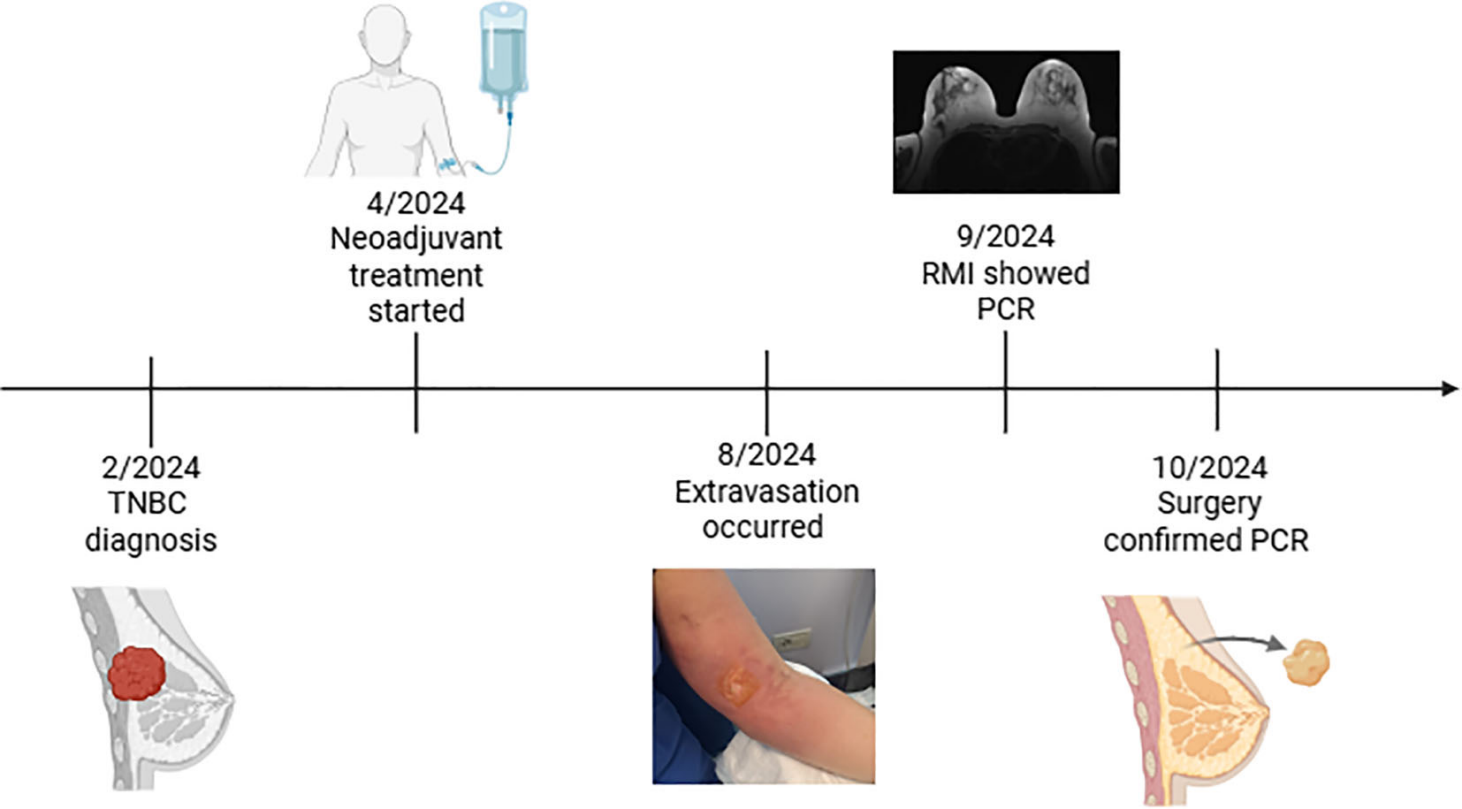
Oncological history timeline.

In September 2024, the patient underwent right mastectomy with sentinel lymph node biopsy (SLNB) surgery. The surgical specimen confirmed the pCR (ypT0, ypN0). After surgery, given the resolution of the extravasation-related injury (**Figure 1**), the exceptional treatment response, and the absence of prior immune-related adverse events, the patient initiated the adjuvant phase with pembrolizumab in October 2024. (**Figure 3** report timeline)

## Discussion

KEYNOTE-522 represents the first phase III, prospective, randomized controlled trial demonstrating statistically significant improvements in pCR, EFS and OS with neoadjuvant pembrolizumab plus chemotherapy in patients with early-stage TNBC (2–4). The greatest magnitude of benefit from the addition of pembrolizumab was observed in patients with stage III disease, as was the case for our patient. In our case the occurring of the extravasation of epirubicin during the first administration led to a non-programmed de-escalation of the neoadjuvant treatment to allow proper care and healing of the site: despite receiving a regimen of carboplatin, paclitaxel, and pembrolizumab only in the neoadjuvant setting, pCR was achieved.

This clinical case raises several questions, one of which is whether a de-escalated treatment approach omitting anthracyclines could be considered in patients with TNBC who exhibit a clinical complete response and how to appropriately identify such cases, especially considering that these patients are often young women treated with a curative intent and therefore a long life expectancy, in which long-term side effects could impair both the quality of life and life expectancy itself.

In fact anthracyclines are well known to cause long-term cardiac side effects, including congestive heart failure and cardiomyopathy (6) and secondary leukemia, including acute myeloid leukemia and myelodysplastic syndrome (5). Anthracycline-free regimens of carboplatin and taxane with or without immunotherapy have been investigated as a de-escalating strategy in neoadjuvant treatment of early TNBC demonstrating promising pCR rates (7–9). The phase II clinical trial NeoPACT, which investigate the role of carboplatin and docetaxel plus pembrolizumab every 21 days for 6 cycles in patients who are not eligible for anthracycline-based regimens, showed encouraging results with a pCR rate of 58% (9). Moreover, in this trial, treatment-related adverse events leading to the discontinuation of any trial drug were reported in 12% of patients, which is favourable compared to the 23% observed in the KEYNOTE-522 trial (3). However, all patients in NeoPACT received mandatory myeloid growth factor support with each cycle (9). On the wave of these results, different trials are evaluating the efficacy of anthracycline-free chemotherapy in combination with immunotherapy, such as the SCARLET trial (Clinical trials.gov identifier: NCT05929768).

Contrary to the de-escalation approach, a recent *post-hoc* analysis of the KEYNOTE-522 trial showed superior outcomes in patients who completed the full chemotherapy regimen compared to those who received less than the full course (10). The pCR rate was lower in patients who could not complete full chemotherapy in both treatment groups, raising questions about the feasibility of treatment de-escalation, also considering the lack of predictive biomarkers. However, it should be noted that this analysis does not provide insight into which part of the chemotherapy regimen was omitted (10).

Nonetheless, appropriate patient selection and thorough evaluation of the optimal treatment duration remain essential to limit long-term toxicities (3).

To date there is a lack of biomarkers to guide the selection of TNBC patients who may benefit from a de-escalated approach, highlighting the need to identify responders to immune checkpoint inhibitors in advance. At present TILs density has been demonstrated to be an independent predictor of OS beyond clinicopathologic features and pathologic response in patients with TNBC treated both with anthracycline-free regimens (11) and anthracycline-containing neoadjuvant chemotherapy (12). A /recent real-world study indicates that elevated TIL levels are strongly correlated with pCR rates in 76 TNBC patients who received the KEYNOTE-522 regimen and could potentially serve as a biomarker for guiding treatment selection (13). In literature, a cut-off value of 30% has been proposed to distinguish between TIL-high and TIL-low groups, and this threshold has also been considered appropriate for use in potential chemotherapy de-escalation studies (14, 15). In addition, as suggested by Sharma et al., other immune biomarkers could have a predictive role, such as the expression of genes involved in DNA damage immune response and genes involved in the tumor immune microenvironment (16).

Moreover, research on radiomic signatures derived from multiparametric MRI images obtained during neoadjuvant treatment could predict treatment response in patients with TNBC is ongoing (17). In the future, an adaptive clinical trial, as the I-SPY trial (18), combining imaging and biological biomarkers, could try to stratify patients addressed to neoadjuvant therapy for TNBC selecting those who could safely receive a de-escalation therapy.

In this case our patient achieved a pCR with an anthracycline-free regimen despite baseline low TILs, tumor response was assessed through breast ultrasound, as per clinical practice, rendering unapplicable the use of radiomic signatures. So, while this clinical case reinforces the possibility of de-escalation of anthracyclines in the neo-adjuvant setting, it does not hint at definite biomarkers that could guide the selection of patients. Whether the inflammatory response triggered by epirubicin extravasation enhances immune-mediated antitumor activity remains unclear. Tissue injury induces sterile inflammation through the release of activated T cells and damage-associated molecular patterns (DAMPs), which can initiate adaptive immune responses by exposing previously unrecognized antigens (19–21). Antigens release from dying cancer cells is known to amplify antitumor immunity, largely through adaptive mechanisms (21). Higher densities of tissue-resident T cells within tumors have been associated with improved survival across multiple cancer types and these cells appear to be the first to respond to ICIs (22). Clonotypic expansion of effector-like T cells has been observed not only within tumors but also in adjacent normal tissue and peripheral blood, suggesting continuous replenishment by fresh, non-exhausted cells from sites outside the tumour (23). These finding support sustained activation of the cancer immunity cycle, which may be associated with clinical benefit. However, whether immune effectors generated by acute tissue injury can subsequently recognize tumor epitopes and enhance ICI efficacy remains speculative.While we acknowledge the need for further studies to validate our immune storm hypothesis, this patient’s unique clinical course raises the possibility that unintended immunologic stimulation may have boosted the immune response and led to the observed outcome.

Currently, all published and ongoing clinical trials investigating anthracycline-free regimens use the combination of carboplatin and docetaxel as the experimental arm (9, 24). In the pre-immunotherapy era, this combination was shown to be more effective than carboplatin-paclitaxel, but it was also associated with higher toxicity and lower tolerability (9). In our case, the complete response obtained using carboplatin, paclitaxel and pembrolizumab raises the hypothesis that the introduction of immunotherapy, possibly enhanced by extravasation, may allow for the use of a less toxic chemotherapy backbone while achieving comparable outcomes.

Our case further highlights how maintaining a high level of attention on risks associated with oncological therapy, particularly in patients undergoing treatment with a curative intent, remains crucial. Our patient accepted a neoadjuvant treatment program with the goal of defeating the disease and prolonging her life expectancy. However, she experienced severe side effects that could have resulted in the loss of her arm or, in the worst-case scenario, death due to superimposed infection and sepsis. These complications caused moments of intense fear, compounded by the fear that leaving the disease untreated could have led to relapse and a shift from a curative to a palliative intent. Fortunately, the outcome was positive.

In conclusion, the KEYNOTE-522 trial demonstrated for the first time that the addition of immunotherapy can achieve unprecedented pCR and OS rates in TNBC. This raises the crucial question of whether such outcomes could be obtained with a less aggressive chemotherapy regimen. Our case report suggests that pCR can be achieved in the absence of anthracyclines, however, considering the lack of validated predictive biomarkers, the use of multiple integrated modalities—such as in the I-SPY trial—could enable more accurate monitoring of treatment response and support a safe de-escalation strategy.

## Data Availability

The raw data supporting the conclusions of this article will be made available by the authors, without undue reservation.
